# Emergency department non-invasive cardiac output study (EDNICO): a feasibility and repeatability study

**DOI:** 10.1186/s13049-019-0586-6

**Published:** 2019-03-11

**Authors:** D. McGregor, S. Sharma, S. Gupta, S. Ahmad, T. Godec, Tim Harris

**Affiliations:** 1 Queen Mary University London and Barts Health NHS Trust, London, UK; 2 University of Western Australia School of Medicine and Pharmacology, Perth, Australia; 3 Emergency Department Research Group, Royal London Hospital, London, UK; 4 Faculty of Epidemiology and Population Health, London School of Hygiene and Tropical Medicine, London, UK; 5 Emergency Medicine, Queen Mary University London and Barts Health NHS Trust, London, UK

**Keywords:** Fluid responsiveness, Stroke volume, Ultrasound, Bioreactance, Plethysmography, Sepsis

## Abstract

**Background:**

There is little published data investigating non-invasive cardiac output monitoring in the emergency department (ED). We assessed six non-invasive fluid responsiveness monitoring methods which measure cardiac output directly or indirectly for their feasibility and repeatability of measurements in the ED: (1) left ventricular outflow tract echocardiography derived velocity time integral, (2) common carotid artery blood flow, (3) suprasternal aortic Doppler, (4) bioreactance, (5) plethysmography with digital vascular unloading method, and (6) inferior vena cava collapsibility index.

**Methods:**

This is a prospective observational study of non-invasive methods of assessing fluid responsiveness in the ED. Participants were non-ventilated ED adult patients requiring intravenous fluid resuscitation. Feasibility of each method was determined by the proportion of clinically interpretable measurements from the number of measurement attempts. Repeatability was determined by comparing the mean difference of two paired measurements in a fluid steady state (after participants received an intravenous fluid bolus).

**Results:**

76 patients were recruited in the study. A total of 207 fluid responsiveness measurement sets were analysed. Feasibility rates were 97.6% for bioreactance, 91.3% for vascular unloading method with plethysmography, 87.4% for common carotid artery blood flow, 84.1% for inferior vena cava collapsibility index, 78.7% for LVOT VTI, and 76.8% for suprasternal aortic Doppler. The feasibility rates difference between bioreactance and all other methods was statistically significant.

**Conclusion:**

Our study shows that non-invasive fluid responsiveness monitoring in the emergency department may be feasible with selected methods. Higher repeatability of measurements were observed in non-ultrasound methods. These findings have implications for further studies specifically assessing the accuracy of such non-invasive cardiac output methods and their effect on patient outcome in the ED in fluid depleted states such as sepsis.

**Electronic supplementary material:**

The online version of this article (10.1186/s13049-019-0586-6) contains supplementary material, which is available to authorized users.

## Background

Intravenous fluid therapy aims to improve cardiac output and therefore restore oxygen delivery to hypoperfused organs. However, iatrogenic tissue oedema may occur when too much intravenous fluid is given. This is associated with increased mortality, renal failure and respiratory compromise [1–3]. Fluid resuscitation in the ED is most commonly guided by physiological parameters (heart rate, blood pressure, capillary refill time) and/or biochemical parameters (pH, lactate and metabolic acidosis), but which are neither sensitive or specific for tissue perfusion [4–7].

Fluid responsiveness is commonly defined as a stroke volume increase of at least 10% following a fluid bolus of 200-500mls 10–15 min [8]. Targeting intravenous fluid therapy to fluid responsiveness and fluid tolerance, and therefore stroke volume/cardiac output, may optimise tissue oxygenation and reduce the risk of tissue oedema. In the intensive care setting, fluid resuscitation is frequently guided by invasive monitoring of cardiac output, previously with Pulmonary Artery Catheterisation (PAC), and now commonly with less invasive devices, such as arterial pulse pressure analysis or oesophageal Doppler [9]. However, these methods are invasive and unsuitable for routine monitoring in the Emergency Department [10, 11].

Non-invasive cardiac output monitoring methods which can rapidly identify fluid responsiveness and guide fluid therapy are emerging in the ED and in the pre-hospital environment [12, 13]. These methods include left ventricular outflow tract velocity time integral (LVOT VTI) [14, 15], common carotid artery blood flow monitoring (CCABF) [16], suprasternal aortic Doppler (SSAD) [17, 18], plethysmography using the vascular unloading technique (PVUT) [19, 20], and thoracic bioreactance [21–23]. Inferior vena cava collapsibility index (IVCCI), while not measuring cardiac output, may be an indicator of fluid responsiveness [23–25].

The range of methods available can be confusing to ED practitioners given the absence of systematic comparison. Assessing the clinical value of a diagnostic test is a multi-phase process which includes assessing its feasibility, repeatability, accuracy, impact on patient outcomes, and cost. The objectives of this study were to systematically compare methods for a broad range of ED patients according to two key diagnostic criteria:

1. *Feasibility* defined as the proportion of clinically interpretable results yielded from attempted measurements, and
2. *Repeatability* (or test-retest reliability) defined as the mean difference occurring with repeated measurements of the same target as a marker of internal validity.

## Methods

### Study protocol

**Fig. 1:**
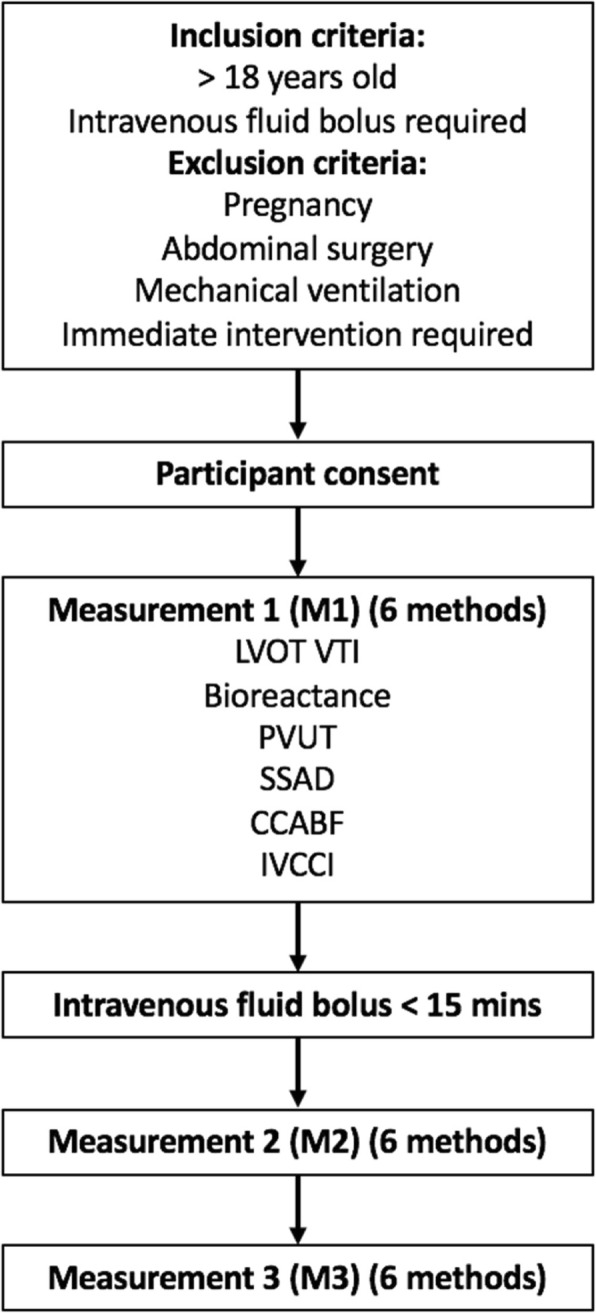
Study participant pathway. SV = stroke volume; CO = cardiac output; PVUT = plethysmography vascular unloading technique; CCABF = common carotid artery blood flow; IVCCI = inferior vena cava collapsibility index; LVOT VTI = left ventricular outflow tract velocity time integral; SSAD = suprasternal aortic Doppler

**Fig. 2:**
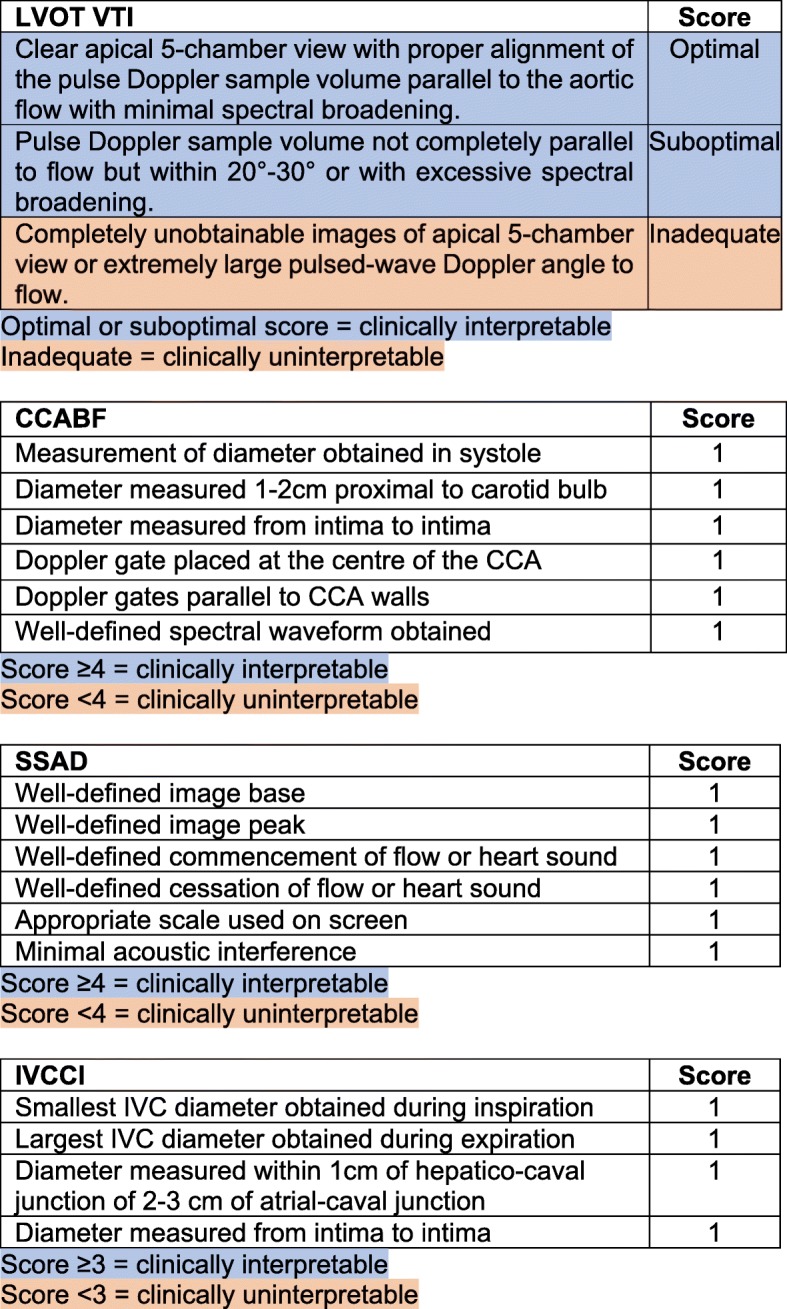
Quality assessment criteria for LVOT VTI, CCABF, SSAD and IVCCI

The study was a prospective observational study. A predefined point score system was used for each technique to sort clinically interpretable measurements from uninterpretable measurements. Feasibility was determined by dividing the number of clinically interpretable measurements by the total number of attempted measurements for each method. Repeatability was determined by comparing the mean difference of two paired measurements in a fluid steady (replete) state for each method (post-fluid measurement rounds, M2 and M3). Participants were placed in a semi-recumbent position at 30 degrees on a trolley. The stroke volume was simultaneously measured by LVOT VTI, CCABF, bioreactance, and PVUT (measurement round 1 - M1). IVCCI was also measured. A 250–500 mls of crystalloid fluid bolus was then delivered over 15 min. A post-fluid measurement round with all six methods was then conducted (M2), immediately followed by a third measurement round (M3) to assess repeatability (Fig. 1). The duration for each measurement was recorded. Ultrasound measurements were saved as screenshots and scored for clinical interpretability by a blinded operator according to a pre-defined pragmatic dichotomous outcome: clinically interpretable versus clinically uninterpretable (Fig. 2). Dinh et al’s method [26] was used to measure stroke volume by LVOT VTI. Stolz et al’s method was used to measure CCABF [27]. The Fremantle criteria [28] were used to measure stroke volume by SSAD. IVCCI was measured in B mode with the minimal and maximal IVC diameters during respiration measured 1 cm distal to the hepatic-caval junction or 2-3 cm distal to the atrial-caval junction as guided by previous studies [24, 25, 29, 30]. IVCCI was calculated with the following formula:

$$ \frac{IVC\  diameter\ (expiration)- IVC\  diameter\ (inspiration)}{IVC\  diameter\ (expiration)} $$

Stroke volume by PVUT and bioreactance was measured following the manufacturer’s instructions. Stroke volume is displayed onto an external monitor for both methods. Feasibility was the proportion of readings displayed as a proportion of all attempted readings one minute prior to starting the fluid bolus and within five minutes of the fluid bolus ending. If no values for stroke volume was displayed during these periods a failed attempt was recorded.

### Participants

Potential participants were screened for inclusion and exclusion criteria by the department clinical team only on arrival to the ED to avoid selection bias. All eligible patients were then referred to the research team for consent. The inclusion criteria were: older than 18 years of age and necessitating an intravenous fluid bolus of 250-500mls as assessed by the clinical care team. The exclusion criteria were: pregnancy, recent abdominal surgery (potential for anatomical alteration), invasive or non-invasive mechanical ventilation, and presentations requiring immediate intervention (systolic blood pressure < 80 mmHg including traumatic or cardiogenic shock, and ventricular or supraventricular tachycardia). All patients attending the ED during the study period during the hours of 09:00 to 20:00 Monday to Friday between August to October 2015 were eligible for recruitment. Patients having received fluids in a pre-hospital setting were not excluded from the study.

### Equipment

LVOT VTI was measured with a uSmart 3300 ultrasound system (Terason, Burlington, MA, USA). Carotid Doppler traces were assessed by a Sonosite EDGE (Sonosite, Bothwell, WA, USA). Suprasternal aortic Doppler traces were obtained using the USCOM-1A (Pty Ltd., Coffs Harbour, NSW, Australia). PVUT was assessed with a LiDCO continuous non-invasive arterial pressure device (LiDCO plus and CNAP, LiDCO Ltd., London, UK). Bioreactance was assessed with a Cheetah Medical device (Cheetah Medical, Portland, OR, USA).

### Operator training

A review of the literature showed that without prior experience performing LVOT VTI at a competent level takes 40–50 practice studies [31], CCABF requires 20–25 studies [27], SSAD requires 20–40 studies [18, 28, 32, 33] and IVCCI requires 50 studies [34, 35]. Three operators (DM, SG, SS) with no prior ultrasound experience were trained to operate all six non-invasive monitoring methods during the same pre-study training programme. The programme consisted of the standard UK Level 1 ultrasound course followed by 50 measurements for all four ultrasound methods on volunteers. Bioreactance and PVUT training was provided by the respective manufacturers for 2 h each. This pragmatic training programme ensured that operators’ performance and experience on all methods was identical and achievable by junior residents and ED nurses. No intra-operator or inter-operator variability assessment was performed.

### Statistical analysis

Measurement data were collected onto a REDCap database (Vanderbilt University, Nashville, TN, USA) and analysed with SPSS v24 (IBM, New York City, NY, USA). *P* < 0.05 (two-tailed) was considered statistically significant. 38 participants with a minimum of 2 rounds of measurements each was the required sample size using a previously described power calculation [36]. Descriptive data are presented with means and 95% confidence intervals and medians with inter-quartile ranges as appropriate. Cochran’s Q test with a Bonferroni correction was used to compare feasibility between methods. Limits of agreement (LoA) analysis was used to assess repeatability as previously described [37]. One-sample t-test was used to verify if the difference between paired measurements (M2 and M3) varied significantly from zero. Bland-Altman analysis was performed to compare paired measurements [38]. Linear regression was used to identify the presence of proportional bias through the range of paired measurements.

### Ethics, consent and permissions

This study was approved by the East of England Research Ethics Committee (REC reference 15/EE/0227; IRAS project ID 172012). Informed consent by each subject was required for participation in this study.

## Results

**Fig. 3:**
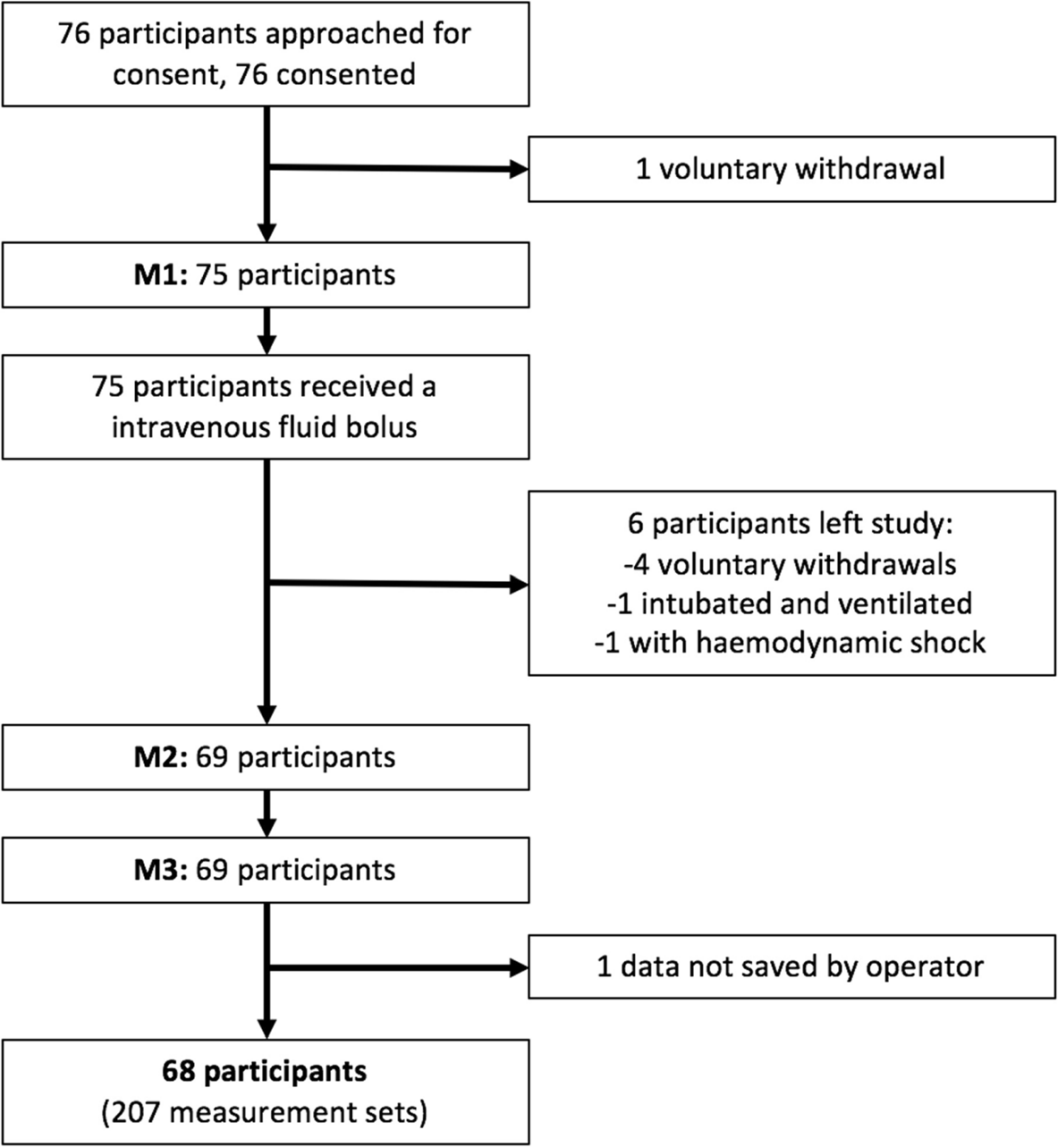
Collection of results

76 participants were recruited in the study including 68 who completed the study protocol (5 voluntary withdrawals, 1 developed signs of shock, 1 was intubated and ventilated, 1 accidental data loss during data transfer) (Fig. 3). Participant characteristics are shown in Table 1.

**Table 1:** Participant baseline characteristics

	Participants (*n* = 76)
Age	52.5 (21.7)
Sex (F:M)	38:38
Body mass index	25.8 (6)
MAP	89.9 (16.8)
SBP	122.5 (22.5)
DBP	69.8 (16)
Heart rate	94.8 (19.4)
Fluid bolus (FB)	373.3 (259.1)
Duration of FB	12.2 (9.9)
Initial lactate	4.74 (8.85)
Previous fluid	390.9 (572.9)
Treated for sepsis (Y:N)	40:36
ED diagnoses*	
**Sepsis**	**40**
Unknown source at presentation	14
Respiratory tract	9
Urinary tract	8
Gastro-intestinal tract	3
Post-operative	3
Cellulitis	1
Dental abscess	1
Neutropenic sepsis	1
**Other**	**36**
Non-specifically unwell	13
Intoxication drugs/alcohol	4
Syncope	4
Viral gastroenteritis	4
Atrial fibrillation > 130 BPM	2
Vestibular neuritis	2
Exacerbation of Crohn’s disease	1
Hyperglycaemic state	1
Ischaemic limb	1
Pulmonary embolism	1
Seizure	1
Renal colic (vomiting)	1
Biliary colic (vomiting)	1

*preliminary diagnosis after initial ED assessment; standard deviations are denoted in brackets, *MAP* mean arterial pressure, *SBP* systolic blood pressure, *DBP* diastolic blood pressure, *ED* emergency department)

### Feasibility

**Fig. 4:**
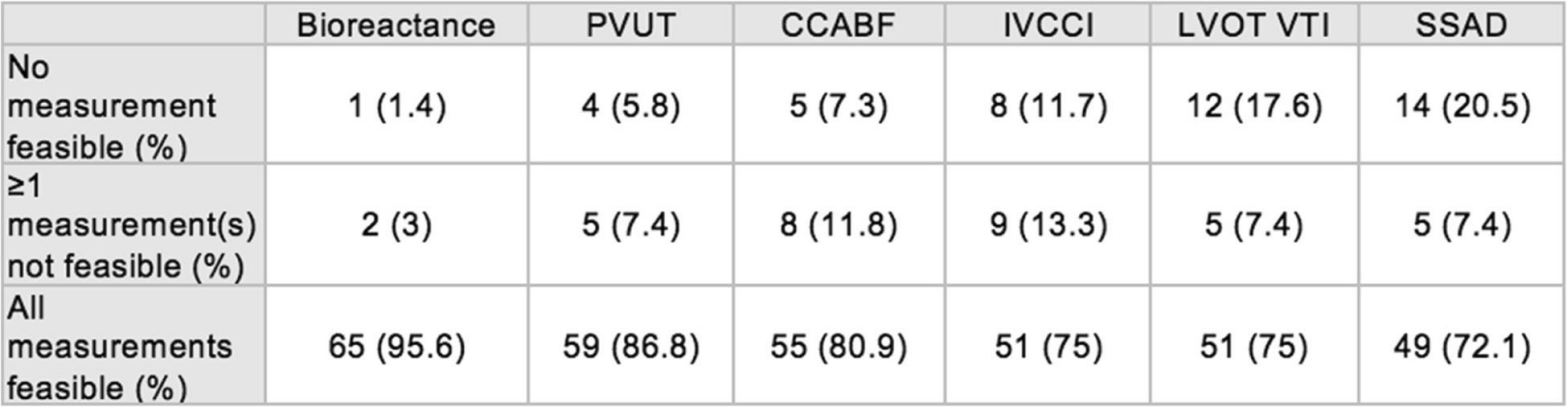
Mean time to completion of M1 for each method (minutes)

Feasibility for the six methods in decreasing order were bioreactance with a completion proportion of 97.6% (202 clinically interpretable results out of 207 attempts; 95% CI: 95.4–99.6%), PVUT with 91.3% (189 clinically interpretable results out of 207 attempts; 95% CI: 87.4–95.1%), CCABF with 87.4% (181 clinically interpretable results out of 207 attempts; 95% CI: 82.9–91.9%), IVCCI with 84.1% (174 clinically interpretable results out of 207 attempts; 95% CI: 79.0–89.0%), LVOT VTI with 78.7% (163 clinically interpretable results out of 207 attempts; 95% CI: 73.1–84.3%) and SSAD with 76.8% (159 clinically interpretable results out of 207 attempts; 95% CI: 71.0–82.5%) (Additional file 1: Fig. S4). Bioreactance had statistically significant superior feasibility to all other methods except PVUT. PVUT was superior to LVOT and SSAD. All other methods were found to have comparable feasibility. Median time to obtaining the first stroke volume measurement (M1) were found to be 0:57 (min:sec) for SSAD, 01:34 for IVCCI, 02:09 for LVOT VTI, 02:26 for CCABF, 07:02 for bioreactance and 08:35 for PVUT (Fig. 4).

### Repeatability

**Fig. 5:**
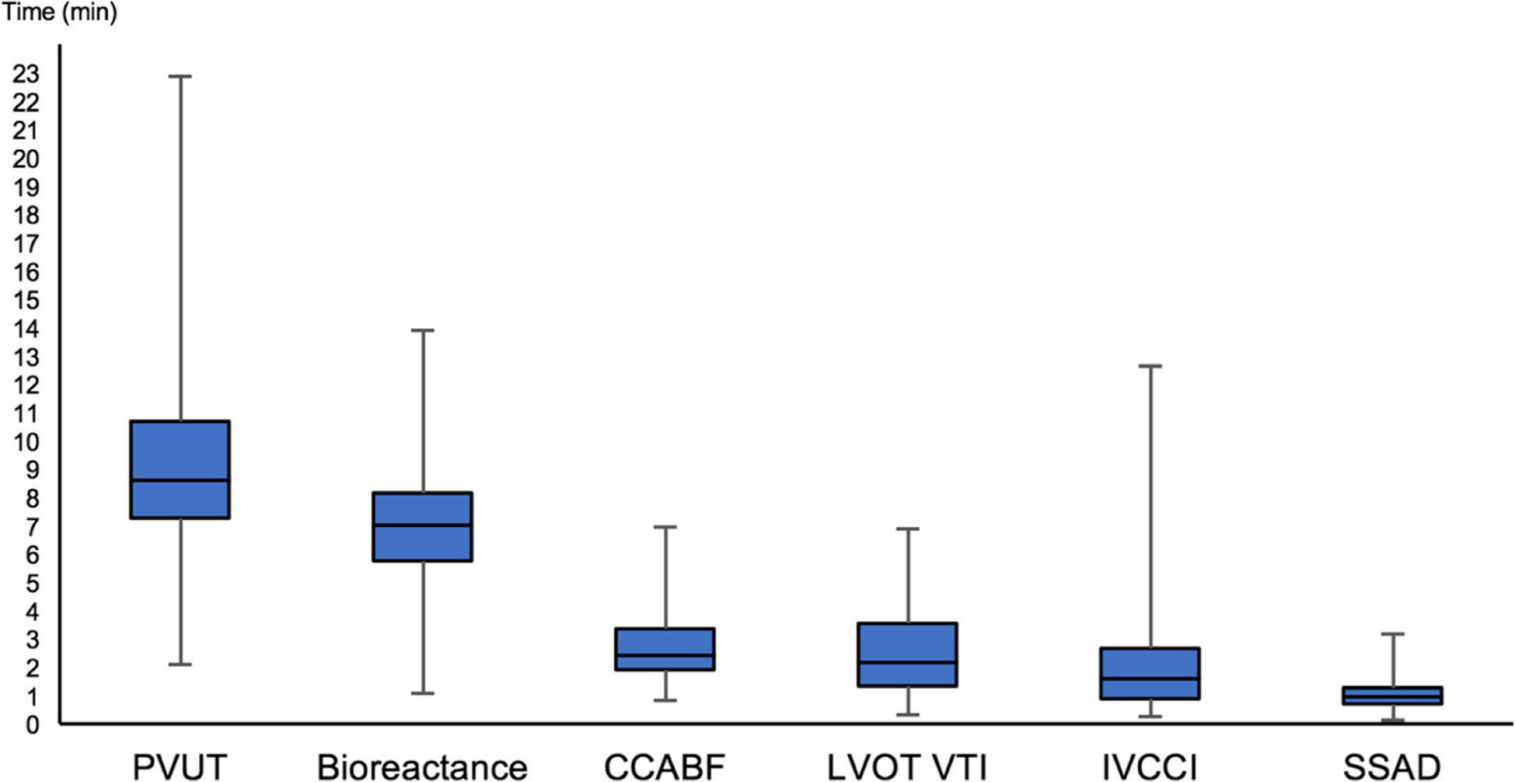
Mean difference between post-fluid measurements M2 and M3

No significant difference was found between post-fluid paired measurements for all six methods (M2 and M3). In other words, after participants received fluids, differences in measurements for M2 and M3 did not significantly vary from zero for each method. Linear regression analysis showed the absence of proportional bias (Additional file 2: Table S2). Bland-Altman plots were traced to define the limits of agreement between paired M2 and M3 measurements (Fig. 5). Clinically acceptable limits of agreement were traced to within +/− 10% of the mean of paired measurements (blue area in Bland Altman plots).

## Discussion

This study shows that the feasibility for assessing fluid responsiveness non-invasively in the ED was greater than 75% for all methods but with significant differences observed between methods. Operator-independent methods (bioreactance and PVUT) showed higher feasibility rates over ultrasound methods. This can be explained by the former requiring less cooperation on part of at times agitated and confused patients and therefore more successful in obtaining measurements. The high feasibility of bioreactance has been reported in trauma patients [39, 40]. PVUT showed significantly higher feasibility than LVOT and SSAD but not CCABF or IVCCI.

The results also illustrate the impact that patient anatomy, care setting and operator skill may have on feasibility for ultrasound methods. For IVCCI, previous studies have reported feasibility rates of 85 to 93% stating notably that adipose tissue and bowel gas can interfere with IVC visualisation [41]. Similarly, in the hands of expert sonographers in ICU, LVOT VTI feasibility ranges from 60 to 100% [42]. However, in ED patients, Dinh et al. found that two ED physicians with 20 h of hands-on instruction obtained a feasibility rate of 78.4% [26], similar to was observed in our cohort of participants. For SSAD, previous studies have reported that tracheostomies, short necks, and thick sternums can limit feasibility with rates of 77 and 87% cited in ICU and ED patients respectively [43, 44]. For CCABF, calcified arterial plaques and cervical arthritis have been described as limiting patient factors [45]. In this study however, CCABF achieved a feasibility rate of 87.4%, the highest among ultrasound methods but without achieving statistical significance.

The absence of standardised measurement technique for IVCCI and CCABF is a concern. A review of IVCCI shows that eight different measurement techniques were used in ten separate diagnostic studies [24, 25, 41, 46–52]. A consensus on standardised technique should be agreed with ED patients in mind; such an approach would ensure continuity of monitoring from arrival and throughput the patient journey in the hospital.

With regards to obtaining a first reading (M1), all ultrasound methods had a median measurement time of under 2 min 30 s (Fig. 4). By contrast, bioreactance and PVUT had median measurement times of over 7 min due to calibration sequences. Inadvertent finger motion by less cooperative participants was found to interfere with PVUT calibration. In one case, a measurement time of 22 min 52 s to obtaining the first reading was recorded. Once calibrated however PVUT and bioreactance both provide continuous cardiac output monitoring.

If feasibility is an essential aspect of clinical value, repeatability informs on the internal validity of the method and therefore the actual quality of the data obtained. This study found no statistically significant differences between the post-fluid paired measurements (M2 and M3) for all methods; each method was therefore able to consistently provide comparable repeat readings in the 95% limits of agreement. To verify if the fluid responsiveness threshold was crossed on paired measurements in a presumably fluid-steady state we applied limits of agreement of +/− 10% to the paired measurement mean. We found that CCABF and IVCCI showed least agreement with most paired readings falling outside these limits. This has important implications. It is likely that operators may require more than 50 scans of baseline experience on CCABF and IVCCI to achieve intra-operator variability of less than 10% to correctly identify fluid responsive states with those methods. It also possible that these methods may also have lower intrinsic repeatability.

### Study limitations

Firstly, it is likely that the absolute feasibility of each technique would be higher if it had been assessed on its own. The higher rates of feasibility generally observed in ICU studies and the longer measurement times seem to support this. This should not affect the relative feasibility between methods. Secondly, repeatability of each method was assessed by paired readings of a fluid steady state (M2 versus M3) rather than paired readings of a fluid dynamic state (change from M1 to M2 versus change from M1*bis* to M2*bis*). This was the preferred approach in view of the time available for measurements. Thirdly, not all participants may have reached a fluid steady state after receiving a fluid bolus of 250-500mls. However, such volume was felt suitable to reverse at least partially fluid depletion in eligible patients.

## Conclusions

The findings of this prospective observational have important implications in helping select suitable method to assess fluid responsiveness in fluid depleted spontaneously breathing ED patients, including septic patients. In our cohort of participants operator-independent methods such as bioreactance and PVUT had high feasibility rates than ultrasound-based methods. The value of such methods should next be compared on their accuracy in identifying fluid responders and their impact on patient outcomes.

## Additional files

Additional file 1: **Figure S4.** Percentage feasibility for non-invasive cardiac output monitoring methods by participant (*n* = 68). Figure illustrating the percentage feasibility for non-invasive cardiac output monitoring methods by participant. (DOCX 132 kb)

Additional file 2: **Table S2.** Proportional bias analysis using linear regression analysis. Table detailing linear regression analysis. (DOCX 13 kb)

## Abbreviations

CCA: Common carotid artery
CCABF: Common carotid arterial blood flow
CNAP: Continuous non-invasive arterial pressure
ED: Emergency department
ICU: Intensive Care Units
IVC: Inferior vena cava
IVCCI: Inferior vena cava collapsibility index
LVOT VTI: Left ventricular outflow tract velocity time integral
PVUT: Plethysmography with vascular unloading technique
SBP: Systolic blood pressure
SSAD: Suprasternal aortic Doppler
USCOM: Ultrasound cardiac output monitor
