# Supplementary figures and images for: Emergency department non-invasive cardiac output study (EDNICO): a feasibility and repeatability study
Source: Scand J Trauma Resusc Emerg Med. 2019 Mar 11;27:30. doi: 10.1186/s13049-019-0586-6 (PMC6417111; doi:10.1186/s13049-019-0586-6)

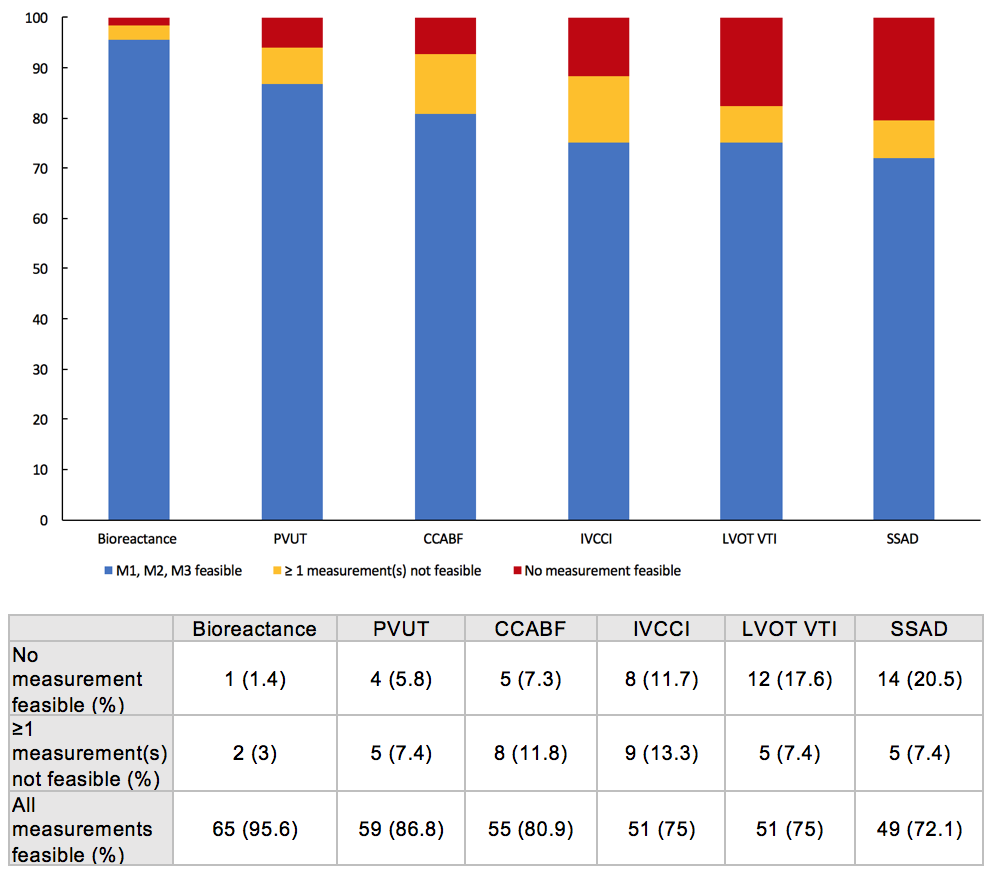


**Additional file 1: Figure S4**.

Supplement: Supplementary file 1 — Figure S4. Percentage feasibility for non-invasive cardiac output monitoring methods by participant (n = 68). Figure illustrating the percentage feasibility for non-invasive cardiac output monitoring methods by participant. (DOCX 132 kb) [file 13049_2019_586_MOESM1_ESM.docx]
