# Supplementary material for: Emergency department non-invasive cardiac output study (EDNICO): a feasibility and repeatability study
Source: Scand J Trauma Resusc Emerg Med. 2019 Mar 11;27:30. doi: 10.1186/s13049-019-0586-6 (PMC6417111; doi:10.1186/s13049-019-0586-6)
Supplement: Supplementary file 2 — Table S2. Proportional bias analysis using linear regression analysis. Table detailing linear regression analysis. (DOCX 13 kb) [file 13049_2019_586_MOESM2_ESM.docx]

Additional file 2: **Table S2.** Proportional bias analysis using linear regression analysis.

| **Method** | **t value** | **p value** |
| --- | --- | --- |
| LVOT VTI | -0.111 | 0.912 |
| CCABF | -0.279 | 0.781 |
| SSAD | 1.087 | 0.283 |
| Bioreactance | 0.615 | 0.541 |
| Plethysmography VUT | 0.249 | 0.804 |
| IVCCI | 0.995 | 0.325 |
